# Identification of Germinants and Expression of Germination Genes in *Clostridium perfringens* Strains Isolated from Diarrheic Animals

**DOI:** 10.3390/pathogens13030194

**Published:** 2024-02-22

**Authors:** Prabhat K. Talukdar, Maryam Alnoman, Mahfuzur R. Sarker

**Affiliations:** 1 Department of Biomedical Sciences, Carlson College of Veterinary Medicine, Oregon State University, Corvallis, OR 97331, USA; 2 Department of Microbiology, College of Science, Oregon State University, Corvallis, OR 97331, USA; 3 Department of Biology, College of Science Yanbu, Taibah University, Al-Madinah 41491, Saudi Arabia; mnaaman@taibahu.edu.sa

**Keywords:** spore germination, amino acids, germinant receptor, cortex hydrolysis

## Abstract

In this study, we investigated the spore germination phenotype of *Clostridium perfringens* strains isolated from diarrheic animals (animal strains). The transcripts of germination-specific genes and their protein products were also measured. Our study found the following results: (i) animal strains spores germinated at a slower rate with AK (mixture of L-asparagine and KCl), L-cysteine, or L-lysine, but the extent of germination varied based on strains and germinants used; (ii) none of the amino acids (excluding L-cysteine and L-lysine) were identified as a universal germinant for spores of animal strains; (iii) animal strain spores germinated better at a pH range of 6.0–7.0; (iv) all tested germination-specific genes were expressed in animal strains; the levels of expression of major germinant receptor gene (*gerKC*) were higher and the cortex hydrolysis machinery genes (*cspB* and *sleC*) were lower in animal strains, compared to the food poisoning strain SM101; and (v) the levels of CspB and SleC were significantly lower in spores of animal strains compared to strain SM101, suggesting that these animal strains lack an efficient spore cortex hydrolysis machinery. In summary, our findings suggest that the poor or slow spore germination in *C. perfringens* animal strains might be due to incomplete spore cortex hydrolysis.

## 1. Introduction

*Clostridium perfringens* is a Gram-positive, anaerobic, spore-forming bacterium that can be found in the environment and in the intestines of humans and animals [1,2]. *C. perfringens* spores germinate and revive into vegetative cells in favorable conditions. Germinated spores produce an array of toxins, which cause a wide range of diseases in humans and animals [3]. The updated toxinotype scheme categorized *C. perfringens* into seven toxinotypes (Type A–G), based on the production of six major toxins [4]. *C. perfringens* is mostly infamous for its enteric disease in humans, but the pathogenicity and disease progression differ among the hosts [5].

For many years, *C. perfringens* has been recognized as an animal pathogen that causes various types of diseases in animals, including livestock and pets [6,7,8]. Most commonly, *C. perfringens* is responsible for necrotic enteritis in birds, horses, cattle, sheep, and pigs, especially in the neonatal individuals of these animals [9,10]. It also causes necrohemorrhagic enteritis in sheep (lamb dysentery) and newborn calves, enterotoxemia in sheep and goats, and animal myonecrosis (gas gangrene) [11,12,13,14]. All these diseases in animals, especially livestock, caused by *C. perfringens* have resulted in a substantial amount of economic loss worldwide. 

*C. perfringens* spores are dormant infectious morphotypes and can withstand various extreme environmental conditions [15,16]. To initiate germination, *C. perfringens* spores must contact germinants (such as amino acids, inorganic salts, etc.) that bind to the germinant receptor (GR) GerKC located in the spores’ inner membrane [17,18]. This is followed by the activation of Csp proteases, which generate SleC, an active cortex-lytic-enzyme (CLE) [19,20,21]. The SleC degrades spore cortex peptidoglycan, leading to the release of monovalent cations (H^+^, Na^+^, and K^+^), and the spore’s core large depot of calcium dipicolinic acid (Ca-DPA) [17]. As a result, the spores’ core uptakes more water from the environment and allows them to resume their metabolic activity [19,22]. 

Vegetative cells are less resistant to environmental cues than their spore counterparts. Therefore, it is easy to kill or destroy *C. perfringens* when the bacterium is in vegetative form. Identifying the effective germinant is crucial for the elimination of *C. perfringens* spores from animal-associated settings and thus minimizes *C. perfringens*-associated diseases in animals. Although numerous studies have been focused on identifying the germinants for the spores of clinical strains originating from humans [18,23,24], very little information has been gathered on the germination of *C. perfringens* strains isolated from diarrheic animals to date. 

In this study, we investigated the germination phenotype of *C. perfringens* strains that were isolated from diarrheic animals (animal strains). The transcripts of germination-specific genes and their protein products were also measured. Our findings suggest that *C. perfringens* animal strains exhibit slow germination compared to human strains and the germination properties differ among the strains. 

## 2. Materials and Methods

### 2.1. Bacterial Strains and Growth Conditions

Six *C. perfringens* strains isolated from diarrheic animals were used in this study. As controls, two well-characterized *C. perfringens* strains of human origin were also included. The relevant characteristics of all *C. perfringens* strains are listed in Table 1. *C. perfringens* strains were maintained in cooked-meat medium (Difco, Becton Dickinson, Sparks, MD, USA) and stored at −20 °C. *C. perfringens* were grown in fluid thioglycolate (FTG) broth (Difco) for vegetative culture; and in Duncan–Strong (DS) sporulation medium (1.5% proteose peptone, 0.4% yeast extract, 0.1% sodium thioglycolate, 0.5% sodium phosphate dibasic, and 0.4% soluble starch) [25] for sporulating cultures. 

### 2.2. Purification of C. perfringens Spores

Different modifications of the DS sporulating medium [25,32] were used to prepare spores from *C. perfringens* animal strains. Spores of *C. perfringens* strains were prepared as previously described [18]. Briefly, *C. perfringens* starter cultures were grown in FTG for overnight at 37 °C, followed by inoculation into freshly prepared 10 mL FTG broth and were incubated for 8 h at 37 °C. Then, 0.4 mL of *C. perfringens* growth culture at log phase was transferred into 10 mL of freshly prepared DS broth and incubated at 37 °C overnight. The presence of spores was confirmed using phase-contrast microscopy, by collecting 10 μL of spores from the sporulating culture and placing them into a glass slide with a cover slip. To scale up the number of spores, similar methods were applied, but in a larger volume. *C. perfringens* cultures grown into three tubes of 10 mL FTG broth were inoculated into 600 mL DS in a large flask and incubated for overnight. Spores were purified by repeated washing with ice-cold sterile distilled water, followed by gradient centrifugation by using 56% Nycodenz (Accurate Chemical and Scientific Corp., Westbury, NY, USA), until spore suspensions were >99% free of cell debris, sporulating cells, and germinating cells, as determined by phase-contrast microscopy. The spore suspensions were adjusted to a final optical density at 600 nm (OD_600_) of ~6.0 with sterile distilled water and stored at −80 °C until used. In the case of a low spore yield for any specific strain, multiple flasks were used to achieve sufficient number of spores for the experiments.

### 2.3. Preparation of Germinant Solutions

Germinant solutions were prepared as described previously [18]. The amino acids that were used in this study were as follows: L-glycine (Gly, MW 75.1 g/mol) (Calbiochem, San Diego, CA, USA), L-alanine (Ala, MW 89.09 g/mol) (EM Science, Gibbstown, NJ, USA), L-valine (Val, MW 117.15 g/mol) (Alfa Aesar, Haverhill, NH, USA), L-leucine (Leu, MW 131.2 g/mol) (California Corporation for Biochemical Research, Los Angeles, CA, USA), L-methionine (Met, MW 149.2 g/mol) (Sigma, St. Louis, MO, USA), L-isoleucine (Ile, MW 131.17 g/mol) (Alfa Aesar), L-proline (Pro, MW 85.0 g/mol) (California Corporation for Biochemical Research), L-phenylalanine (Phe, MW 165.19 g/mol) (TCI America, Portland, OR, USA), L-tyrosine (Tyr, MW 181.2 g/mol) (Sigma), L-tryptophan (Trp, MW 204.23 g/mol) (Sigma), L-serine (Ser, MW 105.1 g/mol) (Research Organics, Cleveland, OH, USA), L-threonine (Thr, MW 119.12 g/mol) (Sigma), L-cysteine hydrochloride monohydrate (Cys, MW 175.63 g/mol) (Sigma), L-asparagine (Asn, MW 132.12 g/mol) (Sigma), L-glutamine (Gln, MW 146.1 g/mol) (Sigma), L-lysine monohydrochloride (Lys, MW 182.65 g/mol) (Sigma), L-arginine (Arg, MW 210.7 g/mol) (Sigma), L-histidine (His, MW 155.15 g/mol) (Sigma), L-aspartic acid (Asp, MW 133.1 g/mol) (Sigma), and L-glutamic acid (Glu, MW 185.3 g/mol) (Sigma). These amino acid compounds were categorized into five groups, according to their side chain (R-group). The germinant solutions were prepared by dissolving the required amount of amino acids in 25 mM Tris-HCl buffer (pH 7.0) to obtain the final concentration of 100 mM, except for L-tyrosine and L-tryptophan; 3 mM of germinant solutions were prepared for L-tyrosine and L-tryptophan, due to their insolubility in Tris-HCl buffer system.

To determine the effect of pH, 100 mM L-cysteine and L-lysine were prepared, mixed in 25 mM Tris-HCl buffer (pH 7.0), and adjusted to various pH levels ranging from 5.0 to 9.0 with 0.5 units increments with 1 M HCl or 1 M NaOH. The concentration effect of L-cysteine and L-lysine was determined by preparing the solutions at various concentrations ranging from 1 mM to 200 mM in 25 mM Tris-HCl (pH 7.0) and adjusted to the final pH of 6.0. 

### 2.4. Spore Germination Assay

Spore germination was carried out as previously described [18,33]. Spore suspensions of OD_600_ of ~6.0 were heat activated [food-poisoning (FP) strain SM101 spores at 80 °C for 10 min; non-foodborne (NFB) strain F4969 spores and all animal strains spores at 75 °C for 15 min] and immediately cooled down at room temperature for 5 min in a water bath followed by warming at 37 °C for 10 min. In a 96-well microtiter plate, heat-activated spore suspensions were mixed with pre-incubated (at 37 °C for 30 min) germinant solutions at a ratio of 1:5 (34 μL spores in 166 μL of germinant solution) to achieve the final spore concentration of an OD_600_ of ~1.0 in 200 μL total volume. Spore germination was routinely measured every 5 min for a 90 min period by monitoring the change in OD_600_ by using a Synergy MX multi-mode microplate reader (BioTek Instrument Inc., Winooski, VT, USA) at 37 °C. Spore germination was further confirmed by phase-contrast microscopy, where the germinated spores looked phase-dark instead of phase-bright for the non-germinated spores. The extent of spore germination was expressed as the percentage of initial OD_600_ values. All germination experiments were performed in duplicate on at least three different occasions, with no fewer than two different individual spore preparations. Standard deviations were calculated from at least six independent measurements. 

### 2.5. Colony Formation Assay 

To evaluate the colony-forming ability of spores of *C. perfringens* strains, spore suspensions of an OD_600_ ~ 1 were heat activated (FP strain SM101 at 80 °C for 10 min and NFB strain F4969 and other animal strains at 75 °C for 10 min) and then cooled to room temperature. The aliquots of serial-diluted heat-activated spores were plated onto brain heart infusion (BHI) agar, incubated anaerobically at 37 °C for 24 h, and colonies counted. Results were expressed as colony forming unit (CFU)/mL/OD_600_.

### 2.6. Extraction of Total RNA and Reverse-Transcription Quantitative Polymerase Chain Reaction (RT-qPCR) Assay

Total RNA was extracted from *C. perfringens*-sporulating cells from 8 h DS broth culture, using the Qiagen RNeasy mini kit (Qiagen, Hilden, Germany). Extracted RNA was digested with DNase I (Thermo Fisher Scientific, Waltham, MA, USA) at 37 °C for 1 h to remove any residual DNA. The cDNA was synthesized with a reverse transcription reaction mixture containing 200 ng RNA and random hexamers, using the iScript cDNA Synthesis Kit (Bio-Rad Laboratories Inc., Hercules, CA, USA). The RT-qPCR was performed in triplicate using iQ SYBR Green Supermix (Bio-Rad Laboratories Inc., Singapore). Each reaction contained a total volume of 10 µl, comprising 5 µL of master mix, 250 nm of each primer, 1 µl of cDNA template, and nuclease-free water. All primers were designed using the Primer 3 web tool (https://primer3.ut.ee/, accessed on 18 February 2024) and are listed in Table 2. The RT-qPCR was performed using CFX96 Real-Time PCR Detection System (Bio-Rad Laboratories Inc., USA) using the following reaction protocol: 95 °C for 3 min, followed by 40 cycles of 95 °C for 10 s, 57 °C for 30 s, and melting curve analysis from 60 °C to 95 °C. Transcript levels were normalized to the housekeeping gene 16s rRNA and calculated by the comparative threshold cycle (Ct) (2^−ΔΔCt^) method. 

### 2.7. Preparation of Spore Extracts and Western Blot Analysis

For preparation of coat extracts from dormant spores, aliquots (200 mL) of spores (OD_600_ of ~30) were decoated in 200 mL of 50 mM Tris-HCl (pH 8.0), 8 M urea, 1% (*w*/*v*) sodium dodecyl sulfate (SDS), and 50 mM 1,4-Dithiothreitol (DTT) for 90 min at 37 °C, centrifuged (13,200 rpm) for 5 min, and the supernatant fluid containing coat material from dormant spores was stored at −20 °C until use. Samples (10 mL) of coat extracts of spores were boiled in sodium dodecyl sulfate-polyacrylamide gel electrophoresis (SDS-PAGE) loading buffer and run on SDS-PAGE gels (12% acrylamide), and proteins were transferred to a polyvinylidene fluoride (PVDF) membrane (Millipore). Western blots were probed with a 1:10,000 dilution of anti-SleC polyclonal rabbit antibody [34] for 1 h at room temperature. For the detection and quantitation of CspB and GerKC, polyclonal rabbit antisera against CspB and GerKC, respectively, was used at a dilution of 1:1000 [34,35]. The PVDF membranes were then incubated with a 1:10,000 dilution of goat anti-rabbit IRDye 800 CW infrared dye-conjugated secondary antibody (LI-COR). The Odyssey Li-Cor Clx (LI-COR) was used to detect secondary antibody fluorescent emissions. The band intensities of the Western blots were quantified using ImageJ software (https://imagej.nih.gov/ij/, accessed on 18 February 2024).

### 2.8. Statistical Analysis

All experiments were performed in triplicate with at least two different batches of spore preparations. Data were analyzed for statistical significance using the GraphPad Prism software v9.0 (GraphPad, La Jolla, CA, USA). A one-way analysis of variance (ANOVA), followed by Dunnett’s multiple comparison test at the significant level of 0.05 (*p* < 0.05), was used to compare the mean and standard deviation (SD) of each group.

## 3. Results

### 3.1. Germination of Spores of C. perfringens Animal Strains with Known Germinants

In our previous studies [18,23,33,36], we showed that the mixture of L-asparagine and KCl (AK), KCl, L-cysteine, and L-lysine significantly induced spore germination in *C. perfringens* type F human FP strain SM101 and NFB strain F4969. We first examined whether these known germinants can induce spore germination in *C. perfringens* animal strains. Spores of different *C. perfringens* strains were incubated with 100 mM KCl (pH 7.0), 100 mM AK (pH 7.0), 100 mM L-cysteine (pH 7.0), 100 mM L-lysine (pH 7.0), or BHI broth (pH 7.0) at 37 °C for 90 min, and germination was measured by monitoring OD_600_ decrease. Germination was also performed with 25 mM Tris-HCl buffer (pH 7.0) as a negative control.

**Figure 1:**
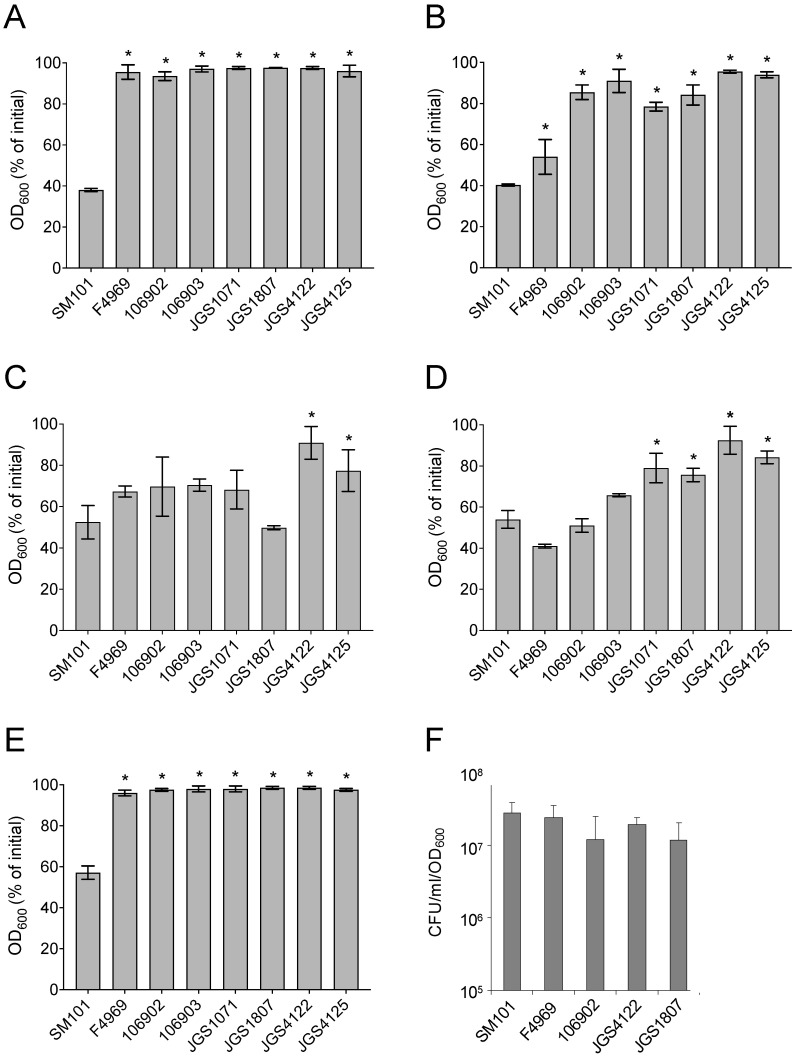
Germination (**A**–**E**) and colony formation (**F**) by spores of *C. perfringens* strains. (**A**–**E**) Heat-activated spores of *C. perfringens* strains were incubated at 37 °C for 90 min with: (**A**) 100 mM KCl (pH 7.0), (**B**) 100 mM AK (pH 7.0), (**C**) 100 mM L-cysteine (pH 7.0), (**D**) 100 mM L-lysine (pH 7.0), and (**E**) BHI broth (pH 7.0), and germination was monitored by measuring OD_600_. The percentage of initial OD_600_ was calculated as described in Material and Methods. (**F**) Colony-forming efficiency of *C. perfringens* spores. Heat-activated spores of *C. perfringens* strains were plated onto BHI agar and colonies were counted after anerobic incubation at 37 °C for 24 h. All values represent the results obtained from at least three individual experiments with two technical replicates in each experiment. Spores were prepared at different times for each individual experiment. The asterisk indicates spores germinated significantly differently, compared to the SM101 strain (* *p* < 0.05). Statistical significance was determined by using a one-way ANOVA followed by Dunnett’s multiple comparison test.

As expected, spores of FP strain SM101 and NFB strain F4969 germinated well with AK, L-cysteine, and L-lysine, but not with Tris-HCl. An ~ 50–60% decrease in OD_600_ was observed after incubation of SM101 or F4969 spores with 100 mM AK (pH 7.0), 100 mM L-cysteine (pH 7.0), or 100 mM L-lysine (pH 7.0) at 37 °C for 90 min (*p* < 0.05) (Figure 1B–D). As consistent with our previous finding [33], while SM101 spores germinated significantly with BHI or KCl, F4969 spores germinated very poorly; only a ~ 10–15% OD_600_ decrease was observed when F4969 spores were incubated with 100 mM KCl (pH 7.0) or BHI (pH 7.0) at 37 °C for 90 min (Figure 1A,E).

When six animal strains spores were incubated with AK, L-cysteine, and L-lysine, the extent of germination varied based on the strains and germinants used. An ~ 15–50% decrease in OD_600_ was obtained after incubation of animal strains spores with 100 mM AK (pH 7.0), 100 mM L-cysteine (pH 7.0), or 100 mM L-lysine (pH 7.0) at 37 °C for 90 min (Figure 1B–D). The most significant germination was observed with L-cysteine followed by L-lysine and AK. However, similar to F4969 spores, animal strains spores germinated very poorly with KCl or BHI, as no significant decrease in OD_600_ values was observed when animal strains spores were incubated with 100 mM KCl (pH 7.0) or BHI (pH 7.0) at 37 °C for 90 min (Figure 1A,E). Interestingly, although animal strain spores exhibited significantly lower germination than SM101 spores, spores of animal strains had a colony-forming efficiency on BHI agar plate similar to that of the SM101 or F4969 spores (Figure 1F). Collectively, these results indicated the following: (i) both L-cysteine and L-lysine are able to induce spore germination in animal strains, although not to the same level as strains SM101 or F4969; (ii) germination phenotype of animal strains is somewhat similar to that of F4969, but not to SM101; and (iii) the animal strain spores are not defective in germination, as these spores eventually lead to CFU titers similar to those of SM101 or F4969 spores.

### 3.2. Germination of C. perfringens Animal Strains Spores with Individual Amino Acids

To identify a germinant that induces significant spore germination in *C. perfringens* animal strains, we performed germination assays using 18 individual amino acids. We examined the germination of spores of different *C. perfringens* strains by incubating spores with individual amino acids at 37 °C for 90 min and monitoring OD_600_ decrease. 

Considering at least a 20% decrease in OD_600_ as significant germination, SM101 spores germinated well with 13 out of 18 amino acids at pH 7.0 (Table 3). In contrast, only two amino acids (L-methionine, L-glutamine) could induce the germination of spores of NFB strain F4969 (Table 3). 

Among the 18 amino acids tested, four amino acids (L-asparagine, L-arginine, L-aspartic acid, and L-glutamic acid) induced spore germination in pig strain JGS1071. However, only a single amino acid could induce spore germination in horse strains 106902 (L-arginine) and 106903 (L-serine). Interestingly, none of the amino acids induced spore germination in pig strain JGS1807, and poultry strains JGS4125 and JGS4122 (Table 3). Collectively, our results indicate that none of the amino acids, other than L-cysteine and L-lysine, can be used as a universal germinant for spores of *C. perfringens* animal strains.

### 3.3. Effect of pH and Concentrations of L-Cysteine and L-Lysine on Germination of Spores of Animal Strains

**Figure 2:**
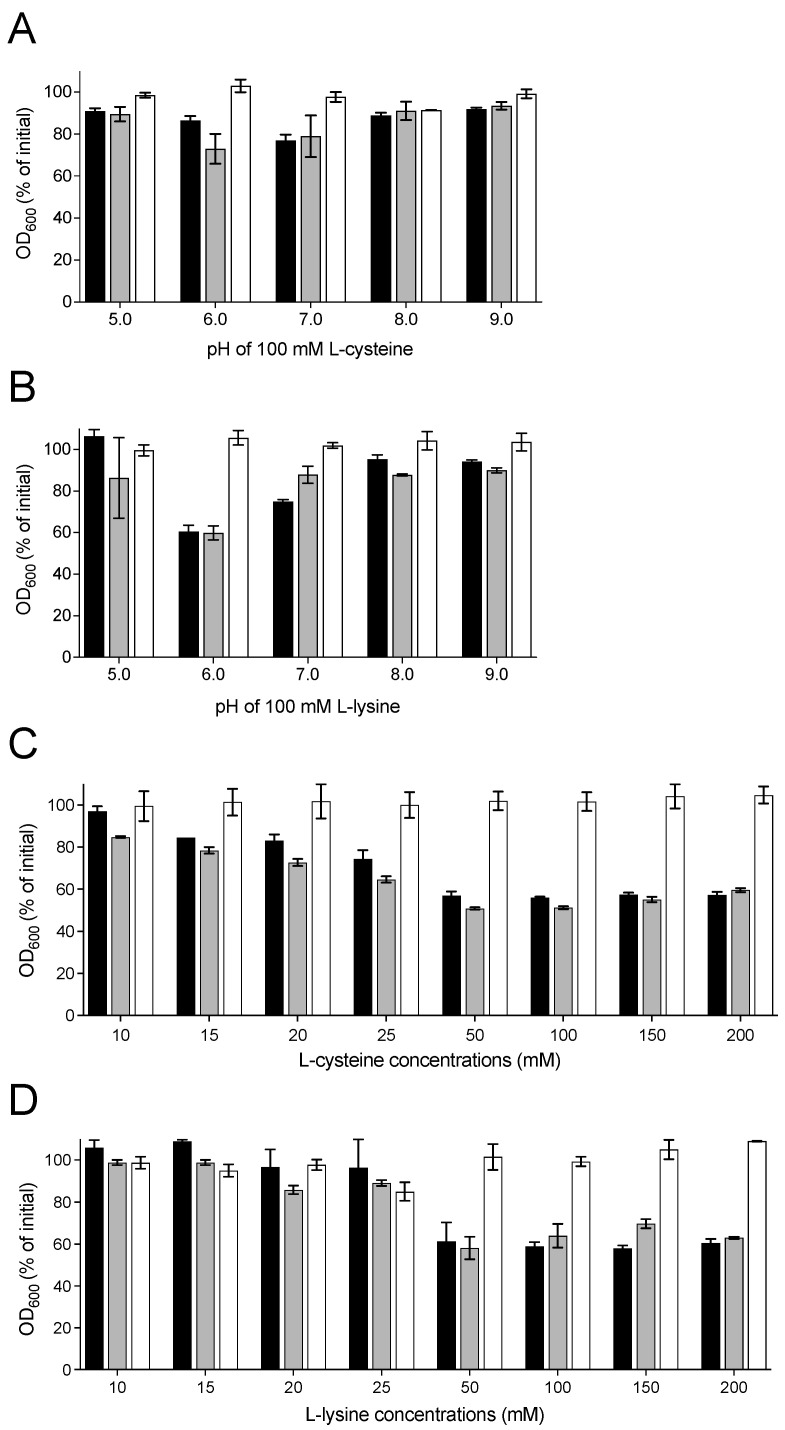
Effect of pH (**A**,**B**) and concentrations (**C**,**D**) of L-cysteine and L-lysine on germination of spores of *C. perfringens* animal strains. (**A**,**B**) Heat-activated spores of horse strain 106903 (black bars), pig strain JGS1807 (grey bars), and poultry strain JGS4122 (white bars) were germinated with 100 mM L-cysteine (**A**) or 100 mM L-lysine (**B**) at various pHs. (**C**,**D**) Heat-activated spores of horse strain 106903 (black bars), pig strain JGS1807 (grey bars), and poultry strain JGS4122 (white bars) were germinated with various concentrations of L-cysteine (pH 6.0) (**C**) or L-lysine (pH 6.0) (**D**). The extent of germination was measured by monitoring a decrease in OD_600_ after 90 min incubation at 37 °C, as described in Material and Methods. At least three separate experiments were performed with two different spore preparations. Error bars represent standard deviations.

As the pH of the germinant solution plays a critical role in inducing *C. perfringens* spore germination [18,23], we determined the optimum pH for L-cysteine- and L-lysine-induced germination of spores of animal strains. We examined spore germination by measuring the decrease in OD_600_ after incubating animal strain spores at 37 °C for 90 min with 100 mM L-cysteine or 100 mM L-lysine at various pHs (pH 5.0–9.0). Our results showed that both L-cysteine and L-lysine were able to induce the germination of spores of pig strain JGS1807 and horse strain 106903 at a pH range of 6.0–7.0 (Figure 2A,B). No significant germination was observed for the spores of any tested animal strains in the presence of 100 mM L-cysteine or 100 mM L-lysine at pH 5.0, pH 8.0, or pH 9.0. However, *C. perfringens* poultry strain JGS4122 spores did not germinate with 100 mM L-cysteine or 100 mM L-lysine at all tested pHs (Figure 2A,B).

To examine the effect of concentrations of L-cysteine and L-lysine on animal strains’ spore germination, we performed germination assays using different concentrations (10–200 mM) of L-cysteine (pH 6.0) and L-lysine (pH 6.0). A minimum of 25 mM L-cysteine (pH 6.0) was required to induce significant spore germination in horse strain 106903 and pig strain JGS1807 after 90 min incubation at 37 °C (Figure 2C). However, spores of strains 106903 and JGS1807 showed maximum germination with 50 mM L-cysteine (pH 6.0). None of the tested cysteine concentrations could induce spore germination in poultry strain JGS4122 (Figure 2C). In contrast, spores of strains 106903 and JGS1807 required at least 50 mM L-lysine (pH 6.0) to induce significant germination after incubation at 37 °C for 90 min (Figure 2D). Like in L-cysteine, JGS4122 spores did not germinate in the presence of any tested concentrations of L-lysine (pH 6.0) (Figure 2D). Collectively, these results suggest that L-cysteine and L-lysine are effective germinants for all tested horse and pig strain spores at a concentration of 50 mM and a pH of 6.0–7.0, with the exception of poultry strain JGS4122 spores.

### 3.4. Expression of Germination-Specific Genes in C. perfringens Animal Strains

**Figure 3:**
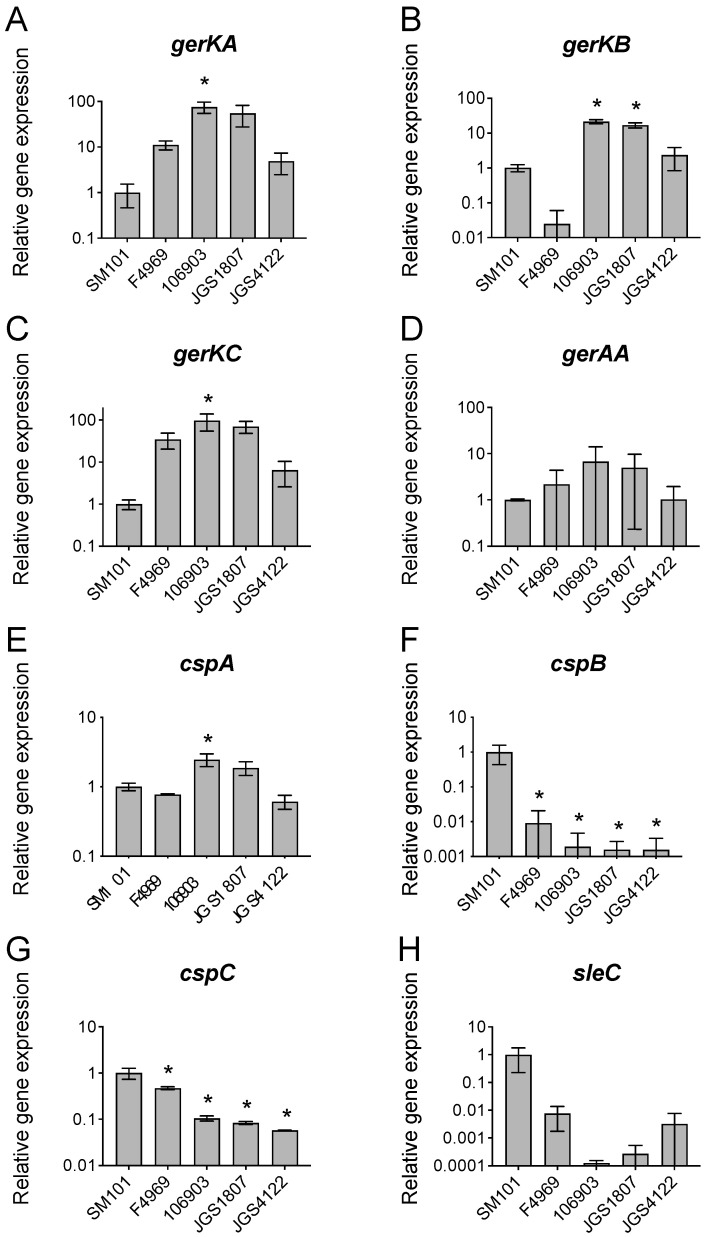
Relative expression of germination genes in *C. perfringens* strains. The total RNA was extracted from the 8 h DS cultures of *C. perfringens* strains as indicated and the cDNA was synthesized and quantitated as described in Material and Methods. The RT-qPCR was performed with cDNA mixed with each set of primers for *gerKA* (**A**), *gerKB* (**B**), *gerKC* (**C**), *gerAA* (**D**), *cspA* (**E**), *cspB* (**F**), *cspC* (**G**), and *sleC* (**H**) genes, and SYBR green qPCR mix and the Cq of the PCR was monitored and recorded. The X-axis denotes the gene expression in different *C. perfringens* strains and the Y-axis denotes the relative gene expression compared to the expression of the housekeeping gene 16s rRNA. Error bars represent standard deviations. The asterisk indicates spores germinated significantly differently compared to the SM101 strain (* *p* < 0.05). Statistical significance was determined using a one-way ANOVA followed by Dunnett’s multiple comparison test.

Having obtained evidence that animal strain spores exhibited slow germination compared to FP strain SM101, we hypothesized that this could be due to the lower expression of germination genes in animal strains. Therefore, we examined the expression of germination-specific genes (GR genes and genes involved in spore cortex hydrolysis, CLE) in animal strains. All tested GR genes are expressed significantly in all *C. perfringens* strains tested. The levels of expressions of four major GR genes (*gerKA*, *gerKB*, *gerKC*, and *gerAA*) were higher in animal strains (106903, JGS1807) than in FP SM101 and NFB F4969 (Figure 3A–D), with the exception of animal strain JGS4122. In contrast to the GR genes, expressions of three CLE genes (*cspB*, *cspC*, and *sleC*) were lower in three animal strains and NFB F4969 compared to that in FP SM101 (Figure 3F–H). Interestingly, *cspA* expression was higher in strains 106903 and JGS1807 and lower in JGS4122 compared to that in FP SM101, respectively (Figure 3E). In summary, these results suggest that animal strains expressed CLE genes at lower levels than the FP strain SM101.

### 3.5. Levels of Germination Proteins in C. perfringens Animal Strains

**Figure 4:**
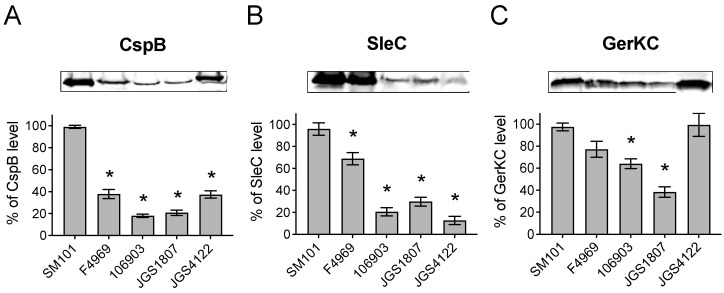
Levels of germination proteins in spores of *C. perfringens* human and animal strains. Spore suspension of OD_600_ of 30 was decoated and was used for Western blot analysis using antibodies against CspB (**A**), SleC (**B**), and GerKC (**C**), as described in Material and Methods. The membrane image was analyzed and quantitated by the ImageJ software. Shown is the representative blot image from two independent experiments. The error bar represents the standard deviation of two independent blot images. The asterisk indicates the significant difference in the protein level of *C. perfringens* strains compared to the SM101 strain (* *p* < 0.05). Statistical significance was determined by using a one-way ANOVA followed by Dunnett’s multiple comparison test.

The lower transcripts of *cle* genes in animal strains suggest the lower production of CLE proteins by these strains. To confirm this, we performed quantitative Western blot analyses to assess the levels of CspB and SleC production in animal strains. We also assessed the production of GerKC, a major GR protein in animal strains. Our results found that the levels of CspB and SleC proteins were significantly lower in spores of animal strains as well as NFB F4969 compared to that in FP SM101 (Figure 4A,B). This finding is in agreement with the low transcript levels of *cspB* and *sleC* in animal strains and NFB F4969 compared to that in FP SM101(Figure 3F,H). Interestingly, an almost similar level of GerKC production was found in poultry strain JGS4122 and FP SM101 as well as NFB F4969 (Figure 4C), although *gerKC* transcript level was significantly higher in JGS4122 than in FP SM101 (Figure 3C). Furthermore, a significantly lower GerKC level was found in strains 106903 and JGS1807 compared to that in FP SM101, although the *gerKC* transcript level was higher in these strains than FP SM101. Collectively, our results suggest that animal strains possess low copies of CLE proteins involved in spore’s cortex hydrolysis compared to the FP strain SM101.

## 4. Discussion

*C. perfringens* is a versatile pathogen that can adapt to different environmental conditions and hosts [3,5]. Therefore, it is likely that *C. perfringens* has a preference for a wide range of germinants. In our previous studies, we observed diversity in the germination requirements between human FP and NFB strains [18,23]. For example, L-asparagine, KCl, a mixture of L-asparagine and KCl (AK), L-cysteine, L-lysine, L-glutamine, and sodium inorganic phosphate (NaPi) were good germinants for FP strains, while AK, L-alanine, L-valine, L-cysteine, L-lysine, and bicarbonate were good germinants for spores of NFB strains [23,24,36,37]. Interestingly, in our current study, we also found diversity in germinant requirements between *C. perfringens* strains from animal and human sources. *C. perfringens* animal strains did not germinate with KCl and BHI, but exhibited slower germination with AK, L-cysteine, or L-lysine compared to human FP strain SM101. The findings of our current study do not match with the results of a recently published paper by Liggins et al. [38], where *C. perfringens* animal strains germinated with L-alanine, L-phenylalanine, and to some extent with L-arginine. The possible reasons for this discrepancy might be as follows: (i) strain differences, as inter-strain variability in germination response has been reported previously [23]; and/or (ii) sporulation condition, as we prepared spores in DS medium, whereas Liggins et al. prepared spores in solid media [37]. 

We also found variations in germination requirements between strains from similar hosts. For example, L-asparagine, L-arginine, L-aspartic acid, L-cysteine, L-glutamic acid, and L-lysine were able to induce spore germination in pig strain JGS1071, while only L-cysteine and L-lysine could trigger significant germination in another pig strain JGS1807. Although not yet proven, this inter-strain difference in spore germination could be due to the variations of key amino acid residues in the binding site of GRs between these strains. In fact, a previous study showed that alterations of a few amino acids in *Bacillus subtilis* GerBA and GerBB receptors altered the germinant requirement from L-alanine to D-alanine [39]. The diversity in germination response between *C. perfringens* animal strains could also be attributed to differential expression and/or absence of some germination genes involved in the response to a particular germinant [40,41]. The differential germination phenotypes between horse, pig, and poultry strains could also come from the adaptation to the ecological niches specialized for each strain for their survival in such environments [17,18,24,42].

In this study, we found that pH played a role in the germination of *C. perfringens* animal strains spores and this pH preference varies among the strains. All *C. perfringens* strains tested in this study germinated better with L-cysteine or L-lysine at pH 6.0 than at pH 7.0. This is in agreement with our previous findings, where pH 6.0 was the optimum for the germination of spores of *C. perfringens* FP and NFB strains with L-cysteine and L-glutamine [23,43].

Previous studies with *Bacillus* species have shown that super-dormant spores (spores that do not usually germinate with nutrients) possess a low copy number of GRs [44]. In this study, when we assessed the expression of major GR gene *gerKC* and the production of GerKC in animal strains, we found that the expression of *gerKC* was higher in animal strains compared to that in FP strain SM101. However, the GerKC levels were lower in all animal strain spores compared to that in SM101 spores. Interestingly, the least germinated strain JGS4122 spores had GerKC level similar to that in SM101 spores. Together, these findings suggest that the slower germination of spores of our tested animal strains was not due to the low copy number of GRs, but possibly due to the defects in other germination machinery.

Finally, the most important finding of this study was that the slow germination ability of animal strain spores could be linked to the inefficient cortex hydrolysis machinery. The CspB, CspC, and SleC are important components of cortex hydrolysis machinery in *C. perfringens* [19,20]. The transcript levels of *cspB*, *cspC*, and *sleC* were significantly lower in animal strain spores compared to the FP strain SM101. This is supported by the low level of CspB and SleC in animal strains than in SM101. The low levels of CspB and SleC indicate that animal strains lack an efficient cortex hydrolysis machinery, and the low germination ability may be linked to incomplete cortex hydrolysis. Further investigation is necessary to establish the germination mechanism in animal strains.

## 5. Conclusions

In summary, this study reports the diversity in the spore germination requirements among *C. perfringens* strains from animal sources. Spores of animal strains exhibited a slower rate of germination compared to that of *C. perfringens* human FP strain SM101, and this might be due to the defects in cortex hydrolysis machinery in these strains. In this study, we also found that L-cysteine (pH 6.0) or L-lysine (pH 6.0) can be used as universal germinants for *C. perfringens* strains. Overall, the present study contributes to our understanding of the germinant specificities of spores of *C. perfringens* animal strains. Further investigation is necessary to understand the molecular mechanism of the germination process in animal strains. 

## Figures and Tables

**Table 1:** *C. perfringens* strains used in this study.

Strains	Host/ Origin	Relevant Characteristics ^1^	References
SM101	Human	Diarrheic FP strain, chromosomal *cpe*+	[26]
F4969	Human	Diarrheic NFB strain, plasmid *cpe*+	[27]
106902	Horse	Diarrheic strain, *cpb2*+, *cpe-*	[11,28]
106903	Horse	Diarrheic strain, *cpb2*+, *cpe-*	[11,28]
JGS1071	Pig	Diarrheic strain, *cpb2*+, *cpe-*	[29,30]
JGS1807	Pig	Diarrheic strain, *cpb2*+, *cpe-*	[29,30]
JGS4122	Poultry	Diarrheic strain, *cpb2*+, *cpe-*	[31]
JGS4125	Poultry	Diarrheic strain, *cpb2*+, *cpe-*	[31]

^1^ FP; food poisoning, NFB; non-foodborne, *cpb2*; gene encoding *C. perfringens* beta-2 toxin, *cpe*; gene encoding *C. perfringens* enterotoxin; +, presence of genes; -, absence of genes.

**Table 2:** Primers used in the RT-qPCR.

Primer Name	Primer Sequence (5’–3’)	Gene
CPP1386	TCTGGGATATGCGCTCTTCT	*cspA*
CPP1387	ATGGTCCTCCTTGCTCCTTT	*cspA*
CPP1388	TAGGGGCGTTGTTAGACCTG	*cspB*
CPP1389	AGAAGAGCGCATATCCCAGA	*cspB*
CPP1390	CTGCAAGAGTGGCAATTCAA	*cspC*
CPP1391	TCCTTTTCCTCTGGTTCAGG	*cspC*
CPP1392	TTCATGACGGGAGACCAAAT	*sleC*
CPP1393	AGTTTCAGGCCACGTTGAAA	*sleC*
CPP1394	GTGGGAGCTGGAATTGCTT	*gerKA*
CPP1395	TCCAAATCGATACTGCACCA	*gerKA*
CPP1396	TCAACCCTAGGTTCTTTGAGG	*gerKB*
CPP1397	TCCATTCTCTACAAAACCACCA	*gerKB*
CPP1398	AGCGGAGGAGCTTTGTTAAA	*gerKC*
CPP1399	GGGTCTTGAGGGTTCATAACTTC	*gerKC*
CPP1400	GCATTAACCATGAGCGAACA	*gerAA*
CPP1401	GCAACATCAGGCCTTTCTGTA	*gerAA*

**Table 3:** Germination of *C. perfringens* spores with individual amino acid at pH 7.0.

Amino Acids ^1^	Decrease in OD_600_ (% Mean ± SD) ^2^							
	Human Strains		Horse Strains		Pig Strains		Poultry Strains	
	SM101	F4969	106902	106903	JGS1071	JGS1807	JGS4122	JGS4125
*Nonpolar*, *aliphatic*								
Gly	38 ± 3.2	4 ± 0.7	5 ± 1.6	4 ± 12.3	16 ± 8.2	3 ± 2.6	1 ± 2.5	0 ± 6.7
Ala	43 ± 5.0	9 ± 0.5	12 ± 4.3	4 ± 1.9	19 ± 10.0	7 ± 5.9	4 ± 2.4	4 ± 7.3
Val	29 ± 2.4	7 ± 0.1	6 ± 3.1	12 ± 5.1	14 ± 7.9	6 ± 2.4	3 ± 4.4	7 ± 7.6
Leu	34 ± 5.2	4 ± 0.7	2 ± 3.5	4 ± 0.8	8 ± 5.4	2 ± 3.9	−3 ± 7.2	5 ± 10.9
Met	26 ± 2.4	21 ± 0.9	2 ± 6.4	3 ± 3.3	13 ± 6.6	3 ± 3.2	3 ± 2.6	7 ± 5.3
Ile	26 ± 1.2	16 ± 0.4	11 ± 12.6	14 ± 2.3	13 ± 7.4	2 ± 5.9	5 ± 16.6	4 ± 6.5
Pro	6 ± 0.6	7 ± 0.6	3 ± 7.5	−3 ± 3.9	13 ± 6.7	5 ± 1.7	1 ± 6.4	−3 ± 8.9
*Aromatic*								
Phe	−3 ± 7.0	6 ± 1.5	−3 ± 5.7	3 ± 1.8	6 ± 4.6	−3 ± 5.5	−2 ± 1.7	−1 ± 2.0
Tyr	12 ± 6.8	5 ± 4.6	1 ± 4.5	0 ± 2.8	11 ± 2.3	0 ± 3.2	−2 ± 3.8	−4 ± 2.7
Trp	−4 ± 3.2	7 ± 2.8	−2 ± 3.5	−3 ± 6.8	8 ± 3.8	0 ± 3.7	−4 ± 6.8	−4 ± 4.7
*Polar*, *uncharged*								
Ser	22 ± 8.9	13 ± 10.5	16 ± 3.9	23 ± 3.8	12 ± 8.2	16 ± 5.3	−2 ± 15.0	4 ± 8.1
Thr	30 ± 0.6	16 ± 2.4	4 ± 11.3	16 ± 7.9	19 ± 7.2	9 ± 7.1	−1 ± 5.9	4 ± 5.3
Asn	17 ± 6.0	9 ± 2.7	19 ± 8.6	2 ± 8.9	21 ± 9.4	9 ± 6.8	−1 ± 5.4	6 ± 10.1
Gln	32 ± 1.6	29 ± 0.1	17 ± 8.5	−1 ± 2.9	19 ± 6.9	3 ± 8.7	−1 ± 5.0	−5 ± 6.4
*Positively charged*								
Arg	41 ± 2.7	1 ± 6.9	20 ± 7.5	−5 ± 1.1	21 ± 7.8	13 ± 15.2	−1 ± 4.8	2 ± 12.5
His	29 ± 6.8	12 ± 2.6	5 ± 8.8	17 ± 2.6	15 ± 4.3	7 ± 6.1	−1 ± 6.7	8 ± 7.7
*Negatively charged*								
Asp	35 ± 0.9	0 ± 0.6	13 ± 9.7	−4 ± 0.6	21 ± 8.0	9 ± 14.2	1 ± 6.2	2 ± 10.7
Glu	31 ± 0.1	9 ± 1.5	10 ± 7.2	−4 ± 8.4	23 ± 10.5	8 ± 20.2	−6 ± 3.1	−5 ± 3.9

^1^ Individual amino acid was dissolved in 25 mM Tris-HCl buffer (pH 7.0) at a final concentration of 100 mM, except for tyrosine (Tyr) and tryptophan (Trp). Tyr and Trp were used at 3 mM in 25 mM Tris-HCl (pH 7.0). ^2^ Germination was calculated as % of OD_600_ decrease using the following formula: $(1-\frac{{\mathrm{OD}}_{600}\;\mathrm{at}\;90\;\min}{{\mathrm{OD}}_{600}\;\mathrm{at}\;0\;\min})\times 100$. The final values shown in the table were derived from at least three independent experiments from two different spore preparations.

## Data Availability

The original data are available upon request.
